# Bacterial Epidemiology of Surgical Site Infections after Open Fractures of the Lower Limb: A Retrospective Cohort Study

**DOI:** 10.3390/antibiotics10121513

**Published:** 2021-12-10

**Authors:** Tracie Joyner Youbong, Astrid De Pontfarcy, Maxence Rouyer, Alessio Strazzula, Catherine Chakvetadze, Clara Flateau, Samir Sayegh, Coralie Noel, Aurélia Pitsch, Abdelmalek Abbadi, Sylvain Diamantis

**Affiliations:** 1 Groupe Hospitalier Sud Ile de France, 77000 Melun, France; astrid.depontfarcy@ghsif.fr (A.D.P.); maxence.rouyer@ghsif.fr (M.R.); alessio.strazulla@ghsif.fr (A.S.); eka.chakvetadze@ghsif.fr (C.C.); clara.flateau@ghsif.fr (C.F.); samir.sayegh@ghsif.fr (S.S.); coralie.noel@ghsif.fr (C.N.); Aurelia.Pitsch@ghsif.fr (A.P.); abdelmalek.abbadi@ghsif.fr (A.A.); sylvain.diamantis@ghsif.fr (S.D.); 2 EA 7380 Dynamic, Université Paris Est Créteil, EnvA, USC ANSES, 94010 Créteil, France

**Keywords:** open fractures, surgical site infections, bacterial epidemiology

## Abstract

Open lower limb fractures are common injuries, and the occurrence of infectious complications after open fractures is a usual problem. The rate of surgical site infections (SSIs) and the nature and resistance of the germs responsible for SSIs remain poorly described. Our aim was to describe the bacterial epidemiology of SSIs after surgical management of an open lower limb fracture. We conducted a retrospective monocentric cohort study from 1 January 2012 to 31 December 2020 based on the analysis of inpatient records in a non-university hospital of Ile de France region. All patients who underwent surgery for an open lower limb fracture were included. A total of 149 patients were included. In our population, 19 (12.7%) patients developed an SSI. Of these 19 patients, the sample was polymicrobial in 9 (47.4%) patients. In 9 (45%) cases, the samples identified a group 3 enterobacteria, Enterobacter cloacae being the main one in 7 (36.9%) cases. Staphylococci were identified in 11 patients, with Staphylococcus aureus in 9 (47.4%). All Staphylococcus aureus were susceptible to methicillin, and all enterobacteria were susceptible to C3G. Among all SSI, 10 (58.8%) contained at least one germ resistant to amoxicillin/clavulanic acid (AMC). The SSIs rate was 12.7%. The main pathogens identified were Enterobacter cloacae and Staphylococcus aureus. The presence of early SSI caused by group 3 Enterobacteriaceae renders current antibiotic prophylaxis protocols inadequate with AMC in half the cases.

## 1. Introduction

Open fractures of the lower extremity are common injuries after high-energy trauma. They require urgent surgeries of Altemeier contamination class 3 or 4 depending on the time to surgery. Altemeier contamination classes 3 and 4 correspond to contaminated and septic surgeries, respectively [1]. Gustilo, in his study, identified the association between certain types of open fractures and the frequency of surgical site infections involving different pathogens [2]. The Gustilo–Anderson classification of open fractures was used to guide prophylactic and preemptive antibiotic therapy. Infectious complications following open fractures are a common issue, with rates ranging from <1% for grade I open fractures to 30% for grade III fractures [3,4,5,6].

The severity of infection, Gustilo classification, smoking, diabetes, time to antibiotic therapy initiation, and shorter duration of antibiotic prophylaxis have been identified as risk factors for the occurrence of postoperative infections [4,7].

Antibiotic prophylaxis for surgery has proven to be effective in reducing surgical site infections [4,8]. Recommendations on this subject are regularly updated to adjust for new specificities of management and the evolution of bacterial resistance.

According to the French recommendations of the Société Française d’Anesthésie Réanimation (SFAR) [1], antibiotic prophylaxis for open Cauchoix fractures stage II and III, corresponding, respectively, to an open fracture with a skin lesion that presents a high risk of secondary necrosis after suturing and an open fracture with non-suturable skin loss opposite or near the fracture site, regardless of the equipment used, is based on the administration of the amoxicillin/clavulanic acid combination. Clindamycin and gentamicin should be initiated as soon as possible in the case of true allergy to penicillin. The English recommendations are similar to the French recommendations, with the use of either amoxicillin/clavulanic acid or cefuroxime [9]. As for the American recommendations, antibiotic prophylaxis for open Gustilo fractures stages I and II are based on the administration of cefazolin or clindamycin in the case of proven allergy to penicillin. For open Gustilo stage III fractures, antibiotic prophylaxis is based on the combination of ceftriaxone and vancomycin or aztreonam and vancomycin in the case of allergy to penicillin [10].

The microorganisms reported in these postoperative infections of open fractures are, depending on the study, staphylococci in proportions varying between 30% and more than 50%, including MRSA (methicillin-resistant *Staphylococcus aureus*), *Enterobacter* spp. (on average 20–30%), *Pseudomonas* spp., and enterococci (56% according to Glass) [11,12,13,14]. Thus, antibiotic prophylaxis recommendations do not consider the risk of surgical-site infections related to environmental bacteria described in some studies with a water contamination mechanism, in particular with *Pseudomonas* spp. [15,16].

The rate, as well as the nature and resistance of the microorganisms involved, for surgical site infections (SSI), remain poorly described, and older studies do not reflect the evolution of bacterial distribution and resistance. The aim of this study was to describe the bacterial epidemiology of surgical site infections following an open fracture surgery of the lower limb.

## 2. Materials and Methods

This study was a monocentric retrospective cohort study based on the analysis of hospital records of patients from a 350-bed non-university hospital in the Ile de France region. In 2018, the department of “Seine et Marne” had a population of 1,412,516 inhabitants, of which 51.6% were women. The age group 24–59 years was more represented (45.8%) [17]. There are 20 short stay establishments in this department, including 11 public hospitals, with a capacity of 2743 beds [18]. The inclusion period ranged from 01 January 2012 to 31 December 2020.

The inclusion criteria were any patient who underwent surgery for an open fracture of the lower limb.

All patients operated on for an open fracture of the lower limb with missing data on the occurrence of surgical site infection were excluded.

This study was conducted in accordance with the Declaration of Helsinki and national and institutional standards. The local institutional review board did not waive the requirement for explicit patient consent due to the retrospective, monocentric nature of the study without the use of personal data.

Data on patient characteristics, laboratory tests and treatment outcomes were collected from software used in daily clinical practice (Sillage v19.0.1.2 and CGM Lab channel 1.20. 33,686) and included: age, sex, comorbidities (diabetes, immunodeficiency, peripheral artery disease of the lower limbs), lifestyle habits (smoking), time to surgery, antibiotic prophylaxis adapted to Gustilo’s classification [2], time to new surgery, time to SSI, and microbiological results.

Antibiotic prophylaxis was prescribed according to the recommendations of the SFAR on the management of fractures [1]. The duration of antibiotic prophylaxis is generally limited to the operative period, sometimes to 24 h, exceptionally to 48 h, and never beyond. An evaluation of the adequacy of antibiotic prophylaxis was carried out retrospectively by analysis of the medical record.

Surgical site infection was defined according to the criteria established by the center for disease control and prevention (CDC) in 1999, which distinguishes between superficial and deep SSI. Diagnostic criteria include the presence of pus, local inflammatory signs, and microbiological documentation or—simply—the surgeon’s clinical judgment [19].

Deep infection diagnosis is based on criteria such as purulent drainage from the deep incision, formation of a deep abscess, dehiscence of the fascia either by infection or on reoperation, or deep infection in the presence of a metal implant around the bone. In addition, the diagnosis of deep or superficial infection was also based on radiological evidence and cultures obtained either during a secondary procedure to treat an infection or from exuding wounds [19].

Antibiotic treatment of SSIs was adjusted according to the antibiotic sensitivity testing between days 5 and 10.

For univariate analysis, categorical variables were analyzed with the chi-square test or Fisher’s exact test. Continuous variables were assessed using the Mann–Whitney/Wilcoxon test and were expressed as the median and interquartile range (IQR). If the missing data were >10%, there was no calculation of relative risk (RR).

Variables with a univariate *p*-value < 0.050 were included in the multivariate analysis. Statistical analysis was performed using EPI INFO Version 7.2.3.1.

## 3. Results

A total of 149 patients that were operated on for open fractures of the lower limb were included, of whom 92 (61.7%) were men with a median age of 48.5 (27–62.5) years. The median BMI was 25.2 kg/m^2^ (21.7–27.8). Among the included patients, 10 (6.7%) were diabetic, and 9 (6%) were immunosuppressed. Smoking was identified in 19 (13%) patients.

The causes of fractures were mainly mechanical falls and motor vehicle accidents (MVA) in 32.3% and 27.6% of cases, respectively. The leg and ankle were the most frequent sites of open fractures in 48.6% and 30.8% of cases.

A total of 10.9% were classified as grade 3 according to the Gustilo classification, and 47.8% were classified as grade 2.

Antibiotic prophylaxis was administered in 85.2% of our population and was compliant in 40.9% of patients. The causes of non-compliance with antibiotic prophylaxis were inadequate duration and timing of prophylaxis, non-compliance of the antibiotic administered. Fourteen (9.4%) patients did not receive antibiotic prophylaxis, and for 8 (5.4%) patients, no information on antibiotic prophylaxis was recorded.

The median time to surgery was less than 1 h (0–24), with a mean time of 9.7 h (standard deviation (SD) 22.4).

In our study population, 19 (12.7%) patients developed a surgical site infection (SSI) according to the Gustilo classification, as follows: 5 patients (26.3%) were classified as Gustilo stage 1, 10 (52.6%) as stage 2, and 4 (21%) as stage 3. The median time to SSI occurrence was 20.5 days (IQR = 11–76). Of these SSIs, 15 patients (88.2%) had deep SSIs and underwent revision surgery with a median delay of 46 days (IQR = 14–78); 2 patients (1.6%) had superficial SSIs. Table 1 describes the characteristics of patients with open lower limb fractures who had a surgical infection and the factors associated with the occurrence of surgical site infection.

Of the 19 patients with SSI, the sample was polymicrobial in 9 (47.4%) patients. In 9 (47.4%) cases, the samples identified a group 3 enterobacteria, with *Enterobacter cloacae* being the main species in 7 (36.9%) cases. Staphylococci were identified in 11 patients, with MSSA (methicillin-susceptible *Staphylococcus aureus*) in 9 (47.4%). All *Staphylococcus aureus* (SA) were susceptible to methicillin, and all enterobacteria were susceptible to third-generation cephalosporins. In two patients, the samples were rendered sterile. Table 2 shows the microbiological results of the surgical site infections.

Of all surgical site infections, 10 (58.8%) contained at least one organism resistant to amoxicillin/clavulanic acid. Factors associated with the occurrence of an SSI involving amoxicillin/clavulanic acid-resistant bacteria are shown in Table 3.

## 4. Discussion

Our study confirms the high frequency of surgical site infections after open fractures of the lower limb, with an overall rate of 12.7%. This result is higher than that of Li’s study, which found an SSI rate of 9.8% [20]. This may be related to the larger population size or the exclusion of superficial SSIs in Li’s study. In contrast, our result is closer to those found by Leonidou and Malhotra in their studies with SSI rates of 13.6% and 13%, respectively [21,22]. Both studies used the same definition of SSI as in our study.

Regarding risk factors for SSI, our study identified diabetes as the only factor associated with the occurrence of surgical site infections. Although not significant, smoking was more frequently found in patients with surgical site infections. These results may be related to the small size of our population. Other risk factors have been previously identified: male gender, smoking, polytrauma, Gustilo grade III classification, and contaminated wounds [4,7,20].

As in Li’s study [20], the time between fracture and surgery was not associated with the occurrence of surgical site infections.

Microbiologically, group 3 enterobacteriaceae, including *Enterobacter cloacae,* were the most frequently found organisms. This result is similar to that found in the Carsenti-Etesse study [12]. Our study shows a difference in the type of bacteria depending on the time of infection: SSIs involving group 3 Enterobacteriaceae are very early, at 13 days postoperative, whereas SSIs involving *Staphylococcus aureus* occur later, at a median of 52 days. We put forward two explanatory hypotheses: the first is related to the pathophysiological mechanism of the infection involving contamination by telluric germs or bacteria of the digestive flora following a strong inoculum from the trauma. The second hypothesis concerns the lack of microbiological efficacy of antibiotic prophylaxis with amoxicillin/clavulanic acid, which is ineffective against group 3 Enterobacteriaceae or waterborne germs, such as *Pseudomonas*. Late MSSA infections could be explained by a secondary mechanism of exogenous wound infection occurring several weeks after surgery and well after antibiotic prophylaxis. This hypothesis is supported by our observation, where surgical site infections with amoxicillin/clavulanic acid-sensitive germs occur after a longer period of time after surgery by exogenous contamination of the scar. Surgical site infections with amoxicillin and clavulanic acid-resistant organisms occur more rapidly after the first operation, possibly due to failure of the initial antibiotic prophylaxis with amoxicillin/clavulanic acid.

Concerning the two patients with sterile samples, two hypotheses were proposed: first, the absence of identification of the germ may be related to the use of an inadequate identification technique; secondly, a previous course of antibiotics could have decapitated the infection resulting in a negative sample. Our study shows, similar to many studies, partial compliance with the recommendations for the administration of antibiotic prophylaxis with an overall compliance rate of 40.9%. However, no association was found between the occurrence of SSI and non-compliance with antibiotic prophylaxis recommendations.

**Figure 1:**
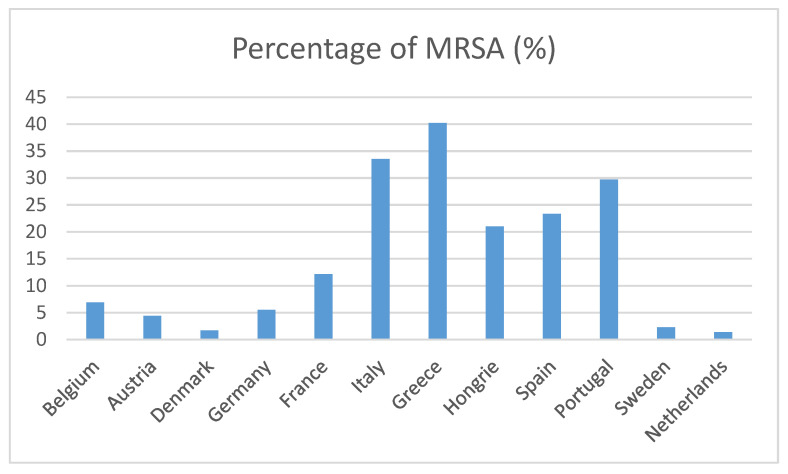
Rate of methicillin resistance in *Staphylococcus aureus* in Europe, 2020 data from EARS-Net ECDC data [23].

Concerning acquired resistance, *Staphylococcus aureus* was always susceptible to methicillin, probably due to the ecology of the study site where methicillin resistance in staphylococci is low, around 15% in France (Figure 1) [23]. Cartenssi’s study [12] reports similar results.

Our study has many limitations. Its monocentric nature leads to a bias related to local epidemiology, which is different according to regions and countries. Our institution is located near the forest of Fontainebleau, where many sports activities at risk of serious trauma, such as horse riding, quad biking, and mountain biking, favor soil contamination. It is likely that centers located in strictly urban areas do not present the same type of trauma.

Our results suggest a failure of the antibiotic prophylaxis strategy with amoxicillin/clavulanate for the prevention of SSI after open limb fractures. These results call for a multicenter prospective cohort study that would allow the confirmation of the results of our study, which would argue in favor of antibiotic prophylaxis with piperacillin-tazobactam in case of land contamination for open fractures.

## 5. Conclusions

The rate of postoperative infections in all open lower limb fractures was 12.7%. The presence of early SSI due to group 3 Enterobacteriaceae suggests that current antibiotic prophylaxis protocols using amoxicillin/clavulanic acid are not effective in half of the cases, warranting a re-evaluation of the protocols and a discussion on the possible use of piperacillin-tazobactam. Our data calls for prospective multicenter studies.

## Figures and Tables

**Table 1:** Description of the population and factors associated with the occurrence of surgical site infection (SSI).

Clinical Characteristics	SSI N = 19 (%)	No SSI N = 130 (%)	RR	*p*
Median age (IQR)	52 (40–67)	47 (26–61)		0.163
Male gender	11 (57.9)	81 (62.3)	0.85 (0.36–1.99)	0.802
Median BMI (IQR) (kg/m^2^) MD ^a^: 86 (>10%)	27.2 (22.1–31.2)	25.2 (21.7–27.4)		0.433
Comorbidities				
Diabetes	4 (21)	6 (4.6)	3.7 (1.51–9.09)	0.024
Immunodeficiency	1 (5.3)	8 (6.2)	0.86 (0.13–5.76)	1
Smoking MD ^a^: 3 (<10%)	4 (21)	15 (11.8)	1.78 (0.66–4.81)	0.276
Fracture site MD ^a^: 3 (<10%)				
Thigh	1 (5.3)	2 (1.6)		0.424
Knee	0	4 (3.1)		
Leg	7 (36.8)	64 (50.4)		
Ankle	6 (31.6)	39 (30.7)		
Foot	5 (26.3)	18 (14.2)		
Gustilo classification MD ^a^: 11				
Stage I	5 (26.3)	52 (43.7)		0.182
Stage II	10 (52.6)	56 (47.1)		
Stage III	4 (21)	11 (9.2)		
Compliant antibiotic prophylaxis				
Yes	12 (70.6)	49 (55.06)	2.5 (1.03–5.9)	0.291
Time to surgery >6 h	5 (38.5)	26 (28.3)	1.5 (0.5–4.2)	0.519
Time to occurrence of SSI (median in days)	20.5 (11–76)			

^a^ missing data.

**Table 2:** Microbiological results of surgical site infections.

Microbiological Results	No. of Cases N = 19 (%)	Time to Infection Median in Days (IQR)
*Enterobacter cloacae*	7 (36.9)	13 (11–54)
Group III enterobacteriaceae	9 (47.4)	13 (11–54)
MSSA	9 (47.4)	52 (13–81)
Polymicrobial	9 (47.4)	13 (13–76)
Resistance to amoxicillin/clavulanic acid	10 (58.8)	13 (11–54)

**Table 3:** Characteristics of SSIs with at least one amoxicillin/clavulanic acid-resistant bacterium.

Microbiological Results	SSI with AMC-Resistant Bacteria N = 10 (%)	SSI with AMC Susceptible Bacteria N = 7 (%)	RR	*p*
Median age (IQR)	66.5 (52–68)	47 (20–66)		0.07
Male gender	6 (60)	3 (42.9)	1.3 (0.6–3)	0.637
Median BMI (IQR) (kg/m^2^)	27.2 (22.4–31.1)	31.5 (22.5–41)	0.643	
Comorbidities				
Diabetes	3 (30)	1 (14.3)	1.4 (0.6–3)	0.603
Immunodepression	1 (10)	0	1.8 (1.1–2.7)	0.588
Smoking	3 (30)	1 (14.3)	1.4 (0.6–3)	0.603
Fracture site				0.249
Thigh	0	0		
Knee	0	0		
Leg	3 (30)	4 (57.1)		
Ankle	3 (30)	2 (28.6)		
Foot	4 (40)	1 (14.3)		
Gustilo classification				
Stage I	1 (10)	3 (42.9)		0.250
Stage II et III	9 (90)	4 (57.1)		
Time to surgery (median in hours)	2 (0–48)	5 (0–17)		0.266
Time to occurrence of SSI (median in Days)	13 (11–54)	54.5 (16–156.5)		0.334

## Data Availability

Data is contained within the article.
